# Metabolic syndrome in children and adolescents - criteria for diagnosis

**DOI:** 10.1186/1758-5996-1-20

**Published:** 2009-10-19

**Authors:** Marcio C Mancini

**Affiliations:** 1 Group of Obesity and Metabolic Syndrome, Endocrinology & Metabolism Service, Faculty of Medicine, University of Sao Paulo, Sao Paulo, Brazil

## Abstract

In recent years, there has been a greater concern about the presence of obesity and metabolic syndrome in children and adolescents. However, there is no consensus regarding the diagnosis of metabolic syndrome in children and adolescents. It is evident that each component of the syndrome must be identified as early as possible in order to prevent definitive lesions. The question is how to do this and which cut-offs must be adopted for this diagnosis. For a matter of convenience, the definition chosen as the most appropriate is the one proposed by the IDF, with cut-offs fixed for pressure, lipids and glycemia, and abdominal circumference points assessed by percentile. Although on the one hand this definition could fail to include some children in the diagnosis of Metabolic Syndrome, on the other hand, it would be of easier acceptance as it does not use multiple tables to assess several anthropometric and metabolic criteria.

## Introduction

We are presently going through a paradox in Brazil expressed by a decrease in infantile malnutrition from 16% in 1974 to 4.6% in 2003 with a gradual increase in obesity, which today occurs in 18% of boys and 15% of girls, according to the Family Budget Research (POF 2002-2003). It is widely known that obesity has been increasing over time and Brazilian data on malnutrition shows that it is decreasing significantly, whereas obesity has been increasing in an opposite way. Nowadays, 18% of boys and 15% of girls are obese[1].

The Bogalusa study, which assessed 66 children autopsies from six years old on with known cardiovascular risk factors, observed that the total cholesterol level and the blood pressure of these children were proportional to the infiltration of foam cells and to the amount of lipids in the arterial intimate layer. Lesions were gradual, evolving from the simple presence of fatty stretch marks to lesions of fibrous plaques and finally to severe lesions characterized by atherosclerotic plaques with fibrosis and necrosis, which have already been observed in children [2].

Evidently, this leads to losing years of life. If the individual's BMI indicates they are overweight from 20 years old on, this would result in losing one year of life in the long term. If we consider the effect of presenting morbid obesity since 20 years of age, this would result in losing 12 years of life in the long term. The later the development of overweight or obesity, fewer are the years of life lost [3]. Therefore, we may infer that it is important for us to treat obesity and metabolic syndrome since childhood.

Several large epidemiologic cohort studies (such as Framingham Offspring Study, Botnia Study, Kuopio Ischemic Heart Disease Study, NHANES II Mortality Study, San Antonio Heart Study and DECODE Study) have documented that metabolic syndrome is associated with cardiovascular outcomes in adults, such as infarction, cerebrovascular disease and sudden death [4]. In recent years, there has been a greater concern about the presence of obesity and metabolic syndrome in children and adolescents. It was demonstrated in 2004 that the prevalence of metabolic syndrome increases as it evolves from a lower tertile to a higher tertile of insulin resistance. This has been observed in different ethnical groups (Caucasians, Hispanics and Blacks), and in different obesity degrees (moderately obese and severely obese individuals). It has also been demonstrated that there is a direct association between insulin resistance tertiles and C-reactive protein, and inverse association with adiponectin levels [5].

## Metabolic Syndrome Diagnosis in Children and Adolescents

There is no consensus regarding the diagnosis of metabolic syndrome in children and adolescents. It is evident that each component of the syndrome must be identified as early as possible in order to prevent definitive lesions. The question is how to do this and which cut-offs must be adopted for this diagnosis.

The diagnosis of metabolic syndrome in children and adolescents requires the assessment of the abdominal circumference (or BMI), pressure, lipoproteins and glycemia. There is even controversy about how to measure the abdominal circumference in adults; measure points differ. Thus, there are proposals: one of them was published in 1999 by Freedman, which is one of the authors of the Bogalusa study [6]. In this article, the authors have correlated the 90^th ^percentile of abdominal circumference with increased levels of LDL cholesterol, glycemia, insulin and diminished HDL levels. Limits were established and the use of a chart for abdominal circumference was proposed (over the 90^th ^percentile, considered to be the maximum normal limit).

By the other hand, Taylor proposed different values. He compared abdominal circumference with abdominal fat measured by densitometry [7]. The possible criticism is that, depending on the age range, the number of children analyzed was very low.

Some people claim that the metabolic syndrome in children must be defined by BMI and not by the abdominal circumference. An adjusted BMI curve according to gender and age was proposed to the Brazilian population, elaborated by the group of Prof. Carlos Monteiro from the Public Health School of University of Sao Paulo (USP) [8]. Despite the existence of this curve even for the matter of comparing studies, BMI curves adjusted by the North-American CDC have been more widely used. The normal BMI varies according to the child's age. Thus, it is impossible to apply a BMI range of 18.5-24.9 to a 12 or 13-year-old child. Overweight is defined from 85^th ^percentile and obesity from 95^th ^percentile [9] (Charts are available at ).

Regarding blood pressure, tables must also be used. Additionally, the inflatable cuff width must be at least 40% of the arm circumference and its length must be at least 80% of the distance between the elbow and the acromion. In practice, it is not necessary to constantly measure the distance, but one must certify that the sleeve occupies most of the arm length circumference. It is recommended to verify the blood pressure of all children from 3 years old on and of children under 3 in case they have associated risk factors [10]

(Charts are available at ).

Regarding lipoproteins, a recent document of the American Academy of Pediatrics establishes the percentiles of total cholesterol, triglycerides, LDL and HDL for boys and girls of several age ranges. Regarding lipoproteins, below the 75^th ^percentile is considered acceptable, corresponding to total cholesterol below 170 mg/dL and LDL below 110 mg/dL. Between 75^th ^and 95^th ^percentile considered borderline and elevated is over 95^th ^percentile. Pharmacological treatment is indicated in the following cases: if risk factors with LDL persistently over 190 mg/dL are not present; if there is any risk factor such as obesity, hypertension or smoking and LDL over 160 mg/dL; and diabetic patients with LDL over 130 mg/dL [11].

Regarding fasting glucose levels, values considered for children and for adults were the same.

## Proposals of Definition of Metabolic Syndrome in Children and Adolescents

The first proposal of definition was published in 2003. It was elaborated by assessing adolescents from 12 to 19 years old using modified criteria, based on the criteria of NCEP/ATP-III, including abdominal circumference over percentile 90, blood pressure over the limits established by the National Blood Pressure Education Program, lipids over the limits established by the National Cholesterol Education Program for children, and glycemia over the values for adults. The general prevalence found in this population of 12-19 years old patients was 4.2%, and when only obese patients over the percentile 95 were considered, the prevalence was 28.7% [12].

The second proposal of definition is very similar to the previous one, but the cut offs were inferior regarding abdominal circumference and lipid profile. Thus, prevalence is higher (when considering patients with BMI percentile adjusted over the percentile 85, it was 31%) [13].

The third proposal chose BMI to serve as a base, justifying that it would be less dependent on ethnical variations - we are aware that abdominal circumference may vary according to the race. The prevalence in moderately obese patients (considering those who had Z of 2 and 2.5) was 38.7%, and in severe obese patients (with Z over 2.5 of pattern deviations), it was 49.7% [14] (Table 1).

**Table 1:** Proposals for rating the metabolic syndrome in children and adolescents

	**Cook *et al.***	**De Ferranti *et al*.**	**Weiss *et al.***
**3 or more among the 5 criteria below**			

Adiposity: abdominal circumference (AC) or BMI	AC ≥ p 90th	AC > p 75th	BMI z score ≥ 2,0
Fasting glycemia or at OGTT (mg/dL)	Fasting gly ≥ 110	Fasting gly ≥ 110	Glycemia at OGTT of 140-200
Blood pressure	≥ p 90th	> p 90th	> p 95th
HDL Cholesterol (mg/dL)	≤ 40	< 50 (girls) e < 45 (boys)	< p 5th
Triglycerides (mg/dL)	≥ 110	≥ 100	> p 95th

Finally, the definition we believe is most appropriate and which was added to this SBD publication is the one proposed by the IDF. It divided children into age groups. There was not a well defined proposal for children under 6 years of age, due to the lack of data. Differently from the criteria presented above, in this proposal, for a matter of convenience, the cut-offs were fixed for pressure, lipids and glycemia, and abdominal circumference points were assessed by percentile. In children aged 6-10, the cut-offs of metabolic and blood pressure variables were not well defined, assessing simply adiposity (considering abdominal circumference over the 90^th ^percentile). The same criteria would be used for children aged 10-16; regarding glycemic metabolism, fasting glycemia ≥100 mg/dL, triglycerides ≥150 mg/dL, HDL cholesterol below 40 mg/dL or using a hypolipemiant drug, and blood pressure limits ≥130 or ≥85 mmHg or using a antihypertensive drug. If the patient had altered abdominal circumference and two more factors, the metabolic syndrome diagnosis would be established. The difference is that, for adolescents over 16 years of age, there is a differentiation between HDL ≤40 for men and ≤50 for women (Table 2) [15].

**Table 2:** IDF proposal for metabolic syndrome definition in children and adolescents

**Criteria/components**	**Age**		
	
	**6 to <10 years-old**	**10 to 16 years-old**	**>16 years-old**
Adiposity definition	WC ≥ 90^th ^percentile	WC ≥ 90^th ^percentile	WC ≥ 90 cm (boys) or ≥ 80 cm (girls)
Glucose metabolism	Without cut-off definition for MS diagnosis	Fasting blood glucose ≥ 100 mg/dl	Fasting blood glucose ≥ 100 mg/dl
Dyslipidemia	Without cut-off definition for MS diagnosis	Tg ≥ 150 mg/dl or HDL-ch ≥ 40 mg/dl or taking LLD	Tg ≥ 150 mg/dl or HDL-ch ≥ 40 (boys) or ≥ 50 mg/dl (girls) or taking LLD
Arterial hypertension	Without cut-off definition for MS diagnosis	DBP ≥ 130 or SBP ≥ 85 mmHg or taking AHD	DBP ≥ 130 or SBP ≥ 85 mmHg or taking AHD

WC: waist circumference; MS: metabolic syndrome; Tg: triglyceride levels; HDL-ch: HDL-cholesterol levels; LLD: lipid-lowering drug; DBP: diastolic blood pressure; SBP: systolic blood pressure; AHD: antihypertensive drug.

Thus, discussions and doubts exist about which criterion to use. Evidently, the IDF criterion, though more convenient, could fail to include some children in the diagnosis of Metabolic Syndrome. On the other hand, it would be of easier acceptance as it does not use multiple tables to assess several anthropometric and metabolic criteria.

## Competing interests

The author declares that he does not have any competing interests regarding the scope of this review.

## Authors' contributions

MCM conceived of the review, including the design and coordination of the text.
